# Blockade of Toll-Like Receptors (TLR2, TLR4) Attenuates Pain and Potentiates Buprenorphine Analgesia in a Rat Neuropathic Pain Model

**DOI:** 10.1155/2016/5238730

**Published:** 2015-12-29

**Authors:** Agnieszka M. Jurga, Ewelina Rojewska, Anna Piotrowska, Wioletta Makuch, Dominika Pilat, Barbara Przewlocka, Joanna Mika

**Affiliations:** Department of Pain Pharmacology, Institute of Pharmacology, Polish Academy of Sciences, 31-343 Krakow, Poland

## Abstract

Accumulating evidence indicates that microglial TLR2 and TLR4 play a significant role in nociception. Experiments were conducted to evaluate the contribution of TLR2 and TLR4 and their adaptor molecules to neuropathy and their ability to amplify opioid effectiveness. Behavioral tests (von Frey's and cold plate) and biochemical (Western blot and qRT-PCR) analysis of spinal cord and DRG tissue were conducted after chronic constriction injury (CCI) to the sciatic nerve. Repeated intrathecal administration of *LPS-RS* (TLR2 and TLR4 antagonist) and *LPS-RS Ultrapure* (TLR4 antagonist) attenuated allodynia and hyperalgesia. Biochemical analysis revealed time-dependent upregulation of mRNA and/or protein levels of TLR2 and TLR4 and MyD88 and TRIF adaptor molecules, which was paralleled by an increase in IBA-1/CD40-positive cells under neuropathy. *LPS-RS* and *LPS-RS Ultrapure* similarly influenced opioid analgesia by enhancing the effectiveness of buprenorphine but not morphine. Summing up, in light of their upregulation over the course of pain, both TLR2 and TLR4 may indeed play a significant role in neuropathy, which could be linked to the observed activation of IBA-1/CD40-positive cells. Blockade of TLR2 and TLR4 produced analgesia and enhanced buprenorphine's effectiveness, which suggests that they may be a putative target for future pharmacological pain relief tools, especially for opioid rotation, when the effect of morphine is tolerated.

## 1. Introduction

Neuropathic pain may appear as a consequence of mechanical nerve injury, the progression of cancer, multiple sclerosis, stroke, and so forth [1, 2]. The currently used analgesics, especially opioid drugs, are not fully effective in reducing chronic pain symptoms [1, 2]; however, the broad range of receptors and signal transduction pathways that could be involved in this process provides a wealth of research opportunities. The current evidence shows that spinal microglia are critically involved in the development and maintenance of neuropathic pain, with a pivotal role of two members of the Toll-like receptor (TLR) family, TLR2 and TLR4 [3, 4]. In the central nervous system, TLR2 and TLR4 are expressed predominantly on glial cells, and for neuropathy, the most relevant expression is on microglia [3, 5].

Direct stimulation of TLRs with exogenous ligands, for example, TLR4 by lipopolysaccharide (LPS), can provoke pain [6]. What is more, induced hypersensitivity is reported to be decreased in TLR2 or TLR4 deficient mice [3, 4]. Most of the proalgesic actions of TLRs are believed to be connected with the detection of pain by sensory neurons in response to local peripheral inflammation [7]. Regarding neuropathic pain, it has been proposed that neuronal damage can lead to the release of proinflammatory factors, for example, saturated fatty acids (SFAs), which activate spinal microglia via the TLR4/NF-kB signaling pathway [8, 9]. Despite numerous studies, the exact functional meaning of both TLR2 and TLR4 for pain as well as the possible differences between them in neuropathic pain remains to be elucidated.

*LPS-RS* (lipopolysaccharide from* Rhodobacter sphaeroides*) is a potent antagonist of TLR2 and TLR4, whereas* LPS-RS Ultrapure* specifically antagonizes TLR4.* LPS-RS* is reported to attenuate hypersensitivity in various neuropathic pain animal models, for example, the Sprague-Dawley rat* paclitaxel*-related chemotherapy-induced peripheral neuropathy (CIPN) model, the cancer-induced bone pain (CIBP) model in Wistar rats, the inflammatory arthritis pain model in* C57Bl/6* mice, and the nerve injury-induced model in Sprague-Dawley rats [10–13]. To our knowledge,* LPS-RS Ultrapure*, a highly specific TLR4 antagonist, has not been used in experiments on animals to date.

What is more, TLR2 or TLR4 deficient animals with induced neuropathy are more resistant to pain [4]. It has been shown that TLR4 activation is mediated by dimerization of adapter proteins such as MyD88 (myeloid differentiation primary response gene 88) or TRIF (TIR-domain-containing adapter-inducing interferon-*β*), but TLR2 uses only MyD88 [14]. Current studies report changes in the protein levels of TLR4 as well as of the MyD88 and TRIF adaptor molecules in pain models (*paclitaxel*-induced neuropathic pain [12] and cancer-induced bone pain [11, 12]); however, as far as we know, their protein levels of TLR4 as well as of the MyD88 and TRIF adaptor molecules have not been studied in neuropathic pain induced by CCI to the sciatic nerve in Wistar rats. Such experiments seem to be important because, in the case of TLR2 and TLR4 regulation, they may show some new mechanisms, which are essential for neuropathic pain development. Recently, it has been shown that TLR2 and TLR4 antagonism produces an analgesic effect in behavioral tests in cancer pain models [11, 12].

Opioid analgesics are commonly used for the treatment of neuropathic pain; however, as has already been mentioned, their efficacy is not satisfactory in comparison to their side effects [15]. In the CNS, microglia play a crucial role in the maintenance of neuronal homeostasis and produce immune factors, which are believed to play an essential role in pain development [16]. It has been shown that, in mice genetically lacking TLR2 or TLR4, microglial activation is markedly decreased, with a parallel reduction of neuropathic pain symptoms [3, 4]. Moreover, it has been reported that antagonism of TLR4 in healthy rats attenuates the development of morphine tolerance [17–20]; therefore, we found it interesting to study how/if TLR2/4 antagonists influence opioid effectiveness in a rat model of neuropathic pain.

Using qRT-PCR and Western blot, we have measured mRNA and protein changes of glial cell markers, TLRs (TLR4 and TLR2), and adaptor molecules (MyD88 and TRIF) in the spinal cord and DRG tissue on the 2nd, 7th, and 14th days after chronic constriction injury of the sciatic nerve in rats. We found it interesting to investigate how* LPS-RS Ultrapure *(a highly specific TLR4 antagonist) and* LPS-RS *(an antagonist of both TLR4 and TLR2) influence neuropathic pain symptoms, such as allodynia and hyperalgesia, which develop after CCI. Another important question which arose is whether these two antagonists of TLRs,* LPS-RS*, and* LPS-RS Ultrapure* might improve the effectiveness of opioids, such as morphine and buprenorphine, in a neuropathic pain model.

## 2. Materials and Methods

### 2.1. Animals

Male Wistar rats (290–330 g) from Charles River (Hamburg, Germany) were housed in cages that were lined with sawdust under a standard 12/12 h light/dark cycle (lights on at 06:00 A.M.), with food and water available* ad libitum*. Care was taken to reduce the number of animals used, and all experiments were performed according to the recommendations of IASP [21] and the NIH Guide for the Care and Use of Laboratory Animals and were approved by the local Bioethics Committee (Krakow, Poland).

### 2.2. Catheter Implantation

Rats were prepared for intrathecal (*i.th.*) injection by implanting catheters according to the method of Yaksh and Rudy [22] under pentobarbital (60 mg/kg;* i.p.*) anesthesia. The intrathecal polyethylene catheter (PE 10, Intramedic; Clay Adams, Parsippany, NJ) was sterilized by immersion in 70% (v/v) ethanol and precisely flushed with sterile water before insertion. Rats were placed on a stereotaxic table (David Kopf Instruments, Tujunga, CA), and an incision was made in the atlantooccipital membrane. The catheter (7.8 cm of its length) was carefully introduced into the subarachnoid space at the rostral level of the lumbar enlargement of the spinal cord (L4-L5), flushed slowly with 10 *μ*L of sterile water, and the tip was tightened. After catheter implantation, the rats were monitored for physical impairments and allowed to recover for a minimum of 1 week before the actual experiment. Animals with visible motor deficits were excluded from further study.

### 2.3. Chronic Constriction Injury (CCI)

CCI was produced in rats according to Bennett and Xie [23] under sodium pentobarbital anesthesia (60 mg/kg;* i.p.*). The* biceps femoris* and the* gluteus superficialis* were separated for right sciatic nerve exposure. Four ligatures (4/0 silk) were tied loosely around the nerve distal to the sciatic notch with 1 mm spacing until they elicited a brief twitch in the respective hind limb. Surgery caused long-lasting neuropathic pain symptoms, such as allodynia and hyperalgesia, in all of the rats.

### 2.4. Drug Administration

*LPS-RS* (a TLR4 and TLR2 antagonist derived from* R. sphaeroides*; InvivoGen, Toulouse, France) and* LPS-RS Ultrapure* (a TLR4-specific antagonist derived from* R. sphaeroides*; InvivoGen, Toulouse, France) were administered at a dose chosen based on the available literature and our preliminary study [10–13, 24].* LPS-RS* [20 *μ*g/5 *μ*L; dissolved in water for injection],* LPS-RS Ultrapure* [20 *μ*g/5 *μ*L; dissolved in water for injection], and vehicle (water for injection) were administered by* i.th.* injection once per day for 9 days (CCI surgery was defined as day 0; substances were administered from day −1 until day 7). The vehicle group received injections (5 *μ*L of water for injection) according to the same schedule. The* i.th.* injections were performed using a 50 *μ*L Hamilton syringe with a 30 1/2-gauge needle; 5 *μ*L was injected per animal, followed by 10 *μ*L of sterile water.

### 2.5. Behavioral Tests

Two behavioral tests, von Frey's and cold plate, were performed at two time points: on 2nd and 7th days after CCI. The tests were conducted in time courses, including the 1st and 3rd hour after the morning drug or vehicle injection.

#### 2.5.1. Mechanical Allodynia (von Frey's Test)

Allodynia was measured in rats subjected to CCI by the use of an automatic von Frey apparatus (Dynamic Plantar Aesthesiometer; Cat. number 37400, Ugo Basile, Italy). The rats were placed in plastic cages with a wire net floor and left for a while to acclimate. The von Frey filament was applied to the midplantar surface of the CCI-exposed ipsilateral and contralateral hind paw, and measurements were taken automatically with a cut-off at 26 g [25].

#### 2.5.2. Thermal Hyperalgesia (Cold Plate Test)

Hyperalgesia was assessed using the cold plate test (Cold/Hot Plate Analgesia Meter; number 05044, Columbus Instruments, USA) as has been described previously [25, 26]. The temperature of the cold plate was maintained at 5°C, and the cut-off latency was 30 s. The rats were placed on the cold plate, and the time until the hind paw was lifted was recorded. The injured paw was the first to react in every case and after animal reaction the animal is taken away from the cage due to minimalized the painful stimulation.

### 2.6. Biochemical Tests

#### 2.6.1. qRT-PCR Analysis of Gene Expression

Ipsilateral dorsal rat spinal cords (L4–L6) were collected on 2nd, 7th, and 14th days after injury. Total RNA was extracted according to the method described by Chomczynski and Sacchi [27] using TRIzol reagent (Life Technologies, Carlsbad, CA, USA) as previously described [28]. RNA concentration was measured using a NanoDrop ND-1000 Spectrometer. Reverse transcription was performed on 1000 ng of total RNA using Omniscript reverse transcriptase (Qiagen Inc., Venlo, Netherlands) at 37°C for 60 minutes. cDNA was diluted 1 : 10 with H_2_O. qRT-PCR was performed using Assay-On-Demand TaqMan probes according to the manufacturer's protocol (Applied Biosystems, Carlsbad, CA, USA) and run on a Real-Time PCR iCycler (Bio-Rad, Hercules, CA, USA). Rn01527838_g1 (*Hprt*), Rn00569848_m1 (*Tlr4*), Rn02133647_m1 (*Tlr2*), Rn01640049_m1 (*MyD88*), Rn02082474_s1 (*Ticam2*), and Rn01423590_m1 (*CD40*) were used as TaqMan primers and probes. Because of disability to design starters based on rat* Trif* sequence, we have used* Ticam2* dedicated primer which, analogically to* Trif*, is connected only to TLR4 and not to TLR2 downstream signaling. The expression of HPRT (a housekeeping gene) was quantified to control for variation in cDNA amounts across groups. Cycle threshold values were calculated automatically by iCycler IQ 3.0 software with the default parameters. RNA abundance was calculated as 2^−(threshold  cycle)^.

#### 2.6.2. Western Blot Analysis

Ipsilateral dorsal lumbar (L4–L6) spinal cord and dorsal root ganglia (DRG) were collected immediately after decapitation on 2nd, 7th, and 14th days after CCI. Tissue was stored at −80°C until processing, which was described previously [28]. Blots were incubated overnight at 4°C with primary antibodies: anti-IBA-1 (rabbit anti-rat, 1 : 1000, Proteintech, Chicago IL, USA), anti-TLR2 (rabbit anti-rat, 1 : 2000, Novus Biological, Littleton CO, USA), anti-TLR4 (rabbit anti-rat, 1 : 1000, Proteintech, Chicago IL, USA), anti-MyD88 (rabbit anti-rat, 1 : 1000, Abcam, Cambridge, UK), and anti-TRIF (rabbit anti-rat, 1 : 500, Novus Biologicals, Littleton CO, USA) and for 1 h at RT with a corresponding secondary polyclonal HRP antibody (goat anti-rabbit IgG, 1 : 5000, Bio-Rad, Hercules, CA, USA). Both primary and secondary antibodies were diluted in solutions from* SignalBoost Immunoreaction Enhancer Kit* (Merck Millipore, Darmstadt, Germany). Immunocomplexes were detected using* Clarity Western ECL Substrate* (BioRad, Hercules, CA, USA) and visualized using a Fujifilm LAS-4000 fluoroimager system. The blots were stripped using* Restore Western Blot Stripping Buffer* (ThermoScientific, Waltham, MA, USA) for 15 minutes at RT and reprobed with an antibody against GAPDH (mouse anti-rabbit, 1 : 5000, Merck Millipore, Darmstadt, Germany) as a loading control.

### 2.7. Data Analysis

*The behavioral data* are presented as the mean ± SEM of 10–25 rats per group. Tests were performed on four groups:* INTACT*,* 2d CCI*: 2 days after injury,* 7d CCI*: 7 days after injury, and* 14d CCI*: 14 days after injury. Intergroup differences were statistically evaluated by ANOVA followed by Bonferroni's* post hoc* test. Significance was defined as ^*∗∗∗*^ *p* < 0.001, indicating a significant difference versus the INTACT group. ^+^ *p* < 0.05, ^++^ *p* < 0.01, and ^+++^ *p* < 0.001 indicate significant differences compared with vehicle-treated CCI-exposed rats; ^##^ *p* < 0.01 and ^###^ *p* < 0.001 indicate a significant difference compared with LPS-RS- or LPS-RS Ultrapure-treated CCI-exposed rats; ^∧^ *p* < 0.05, ^∧∧^ *p* < 0.01, and ^∧∧∧^ *p* < 0.001 indicate differences between opioid-treated CCI-exposed groups.

*The qRT-PCR analyses* from the tissue were performed in four groups:* INTACT*,* 2d CCI*: tissue collected 2 days after injury,* 7d CCI*: tissue collected 7 days after injury, and* 14d CCI*: tissue collected 14 days after injury. The results from 6–8 animals are presented as fold changes compared with the INTACT rats. The qRT-PCR data are presented as the mean ± SEM and represent the normalized averages that were derived from the threshold qRT-PCR cycles from four to eight samples for each group. Intergroup differences were analyzed using ANOVAs followed by Bonferroni's multiple comparison tests. ^*∗*^ *p* < 0.05, ^*∗∗∗*^ *p* < 0.01, and ^*∗∗∗*^ *p* < 0.001 indicate significant differences versus INTACT animals.

*The protein analyses* were performed using the Western blot method. Analysis of the tissue was performed in four groups:* INTACT*,* 2d CCI*: tissue collected 2 days after injury,* 7d CCI*: tissue collected 7 days after injury, and* 14d CCI*: tissue collected 14 days after injury. The results are presented as fold changes compared to the INTACT group. The data are presented as the mean ± SEM and represent the normalized averages derived from analyses of 4–7 samples for each group performed with the Multi Gauge analysis program. Intergroup differences were analyzed using ANOVA followed by Bonferroni's multiple comparison tests. ^*∗*^ *p* < 0.05, ^*∗∗∗*^ *p* < 0.01, and ^*∗∗∗*^ *p* < 0.001 indicate significant differences versus INTACT animals.

## 3. Results

### 3.1. Development of Allodynia and Hyperalgesia due to Neuropathic Pain Development, as Measured on the 2nd, 7th, and 14th Days after CCI

**Figure 1:**
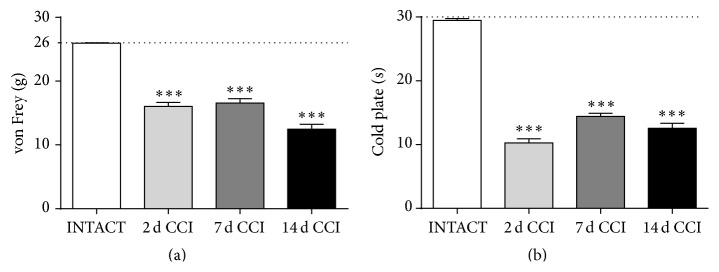
Levels of allodynia ((a); von Frey's test) and hyperalgesia ((b); cold plate test) measured on 2nd, 7th, and 14th days after chronic constriction injury (CCI) in rats. The data are presented as the mean ± SEM (11–25 rats per group). Intergroup differences were analyzed using one-way ANOVA followed by Bonferroni's multiple comparisons test. ^*∗∗∗*^ *p* < 0.001 indicates a significant difference versus the INTACT group.

We observed that mechanical allodynia (von Frey's test) lasted from the 2nd day after injury constantly through the 7th day, reaching a maximum of two weeks after CCI (Figure 1(a)). Thermal hyperalgesia (cold plate test) turned out to be the strongest on day 2 after surgery, which is probably the result of early-stage inflammatory pain, which is silenced until days 7 and 14, when pain is constant and still strong (Figure 1(b)). There was no change as measured at the contralateral paw (25.94 ± 0.6 g) in von Frey test in CCI-exposed rats versus INTACT animals (25.96 ± 0.045 g).

### 3.2. Changes in CD40, TLR2, TLR4, MyD88, and TICAM2 mRNA Levels, as Measured on the 2nd, 7th, and 14th Days after CCI

**Figure 2:**
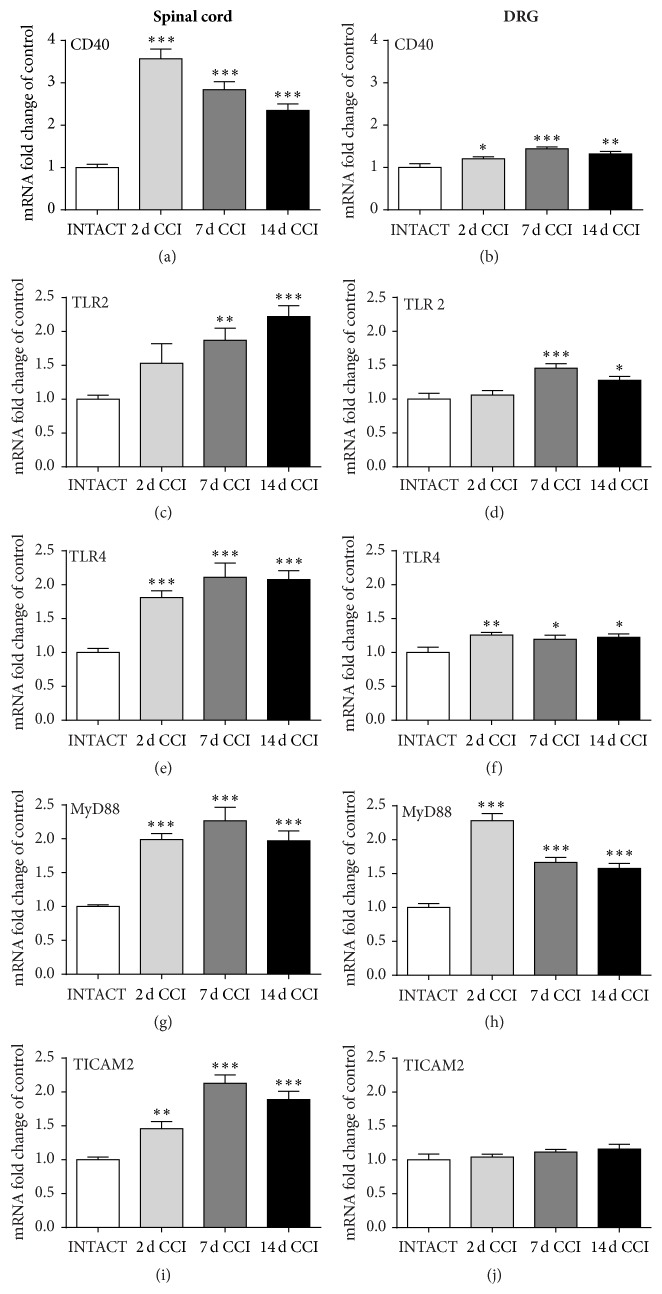
Quantitative real-time PCR analysis of* CD40* (a, b),* TLR2 *(c, d),* TLR4* (e, f),* MyD88* (g, h), and* TICAM2* (i, j) mRNA levels in the ipsilateral dorsal lumbar spinal cord (a, c, e, g, and i) and DRG (b, d, f, h, and j) tissue on 2nd, 7th, and 14th days after chronic constriction injury (CCI) in rats. The data are presented as the mean ± SEM, which represent normalized averages derived from the threshold cycles obtained in qRT-PCR from 6–8 samples per group. Intergroup differences were analyzed using one-way ANOVA followed by Bonferroni's multiple comparisons test. ^*∗*^ *p* < 0.05, ^*∗∗*^ *p* < 0.01, and ^*∗∗∗*^ *p* < 0.001 indicate significant differences versus the INTACT group.

Expression of the marker for CD40-positive cells in the spinal cord had already risen by 237% on 2nd day. Very strong changes, 184% and 135%, were still measured on 7th and 14th days, respectively, after CCI (Figure 2(a)). Weak (21%) upregulation of CD40 was observed on 2nd day in the DRG, the strongest change (44%) on 7th day, and a slight decrease to 32% upregulation on 14th day after CCI (Figure 2(b)).

In contrast, significant changes in TLR2 mRNA were not observed on 2nd day in the spinal cord or DRG. However, a very strong increase in TLR2 mRNA levels was detected on 7th and 14th days, 87% and 122%, respectively, in the spinal cord (Figure 2(c)) and 46% and 28% in the DRG (Figure 2(d)).

The very strong 81% upregulation of TLR4 expression was observed on 2nd day and lasted at a high level (108% of control) until 14th day after CCI in the spinal cord (Figure 2(e)). Changes in the DRG tissue were less pronounced; the strongest change was 26% on 2nd day, which slowly diminished to 22% on 14th day after CCI (Figure 2(f)).

Upregulation of MyD88 expression (99%) was observed until 2nd day, with a peak (127%) on 7th day and lasting at the high level of 97% until 14th day after CCI in the spinal cord (Figure 2(g)). Similar results were obtained in the DRG: the 128% upregulation started on 2nd day, diminished to 67% upregulation on 7th day and to 58% on 14th day after CCI (Figure 2(h)). Significant changes in TICAM2 expression were detected in the spinal cord: 46%, 112%, and 89%, as measured on 2nd, 7th, and 14th days after CCI, respectively (Figure 2(i)). We did not detect any changes in TICAM2 expression in the DRG tissue (Figure 2(j)).

### 3.3. Changes in IBA-1, TLR2, TLR4, MyD88, and TRIF Protein Levels, as Measured on the 2nd, 7th, and 14th Day after CCI

**Figure 3:**
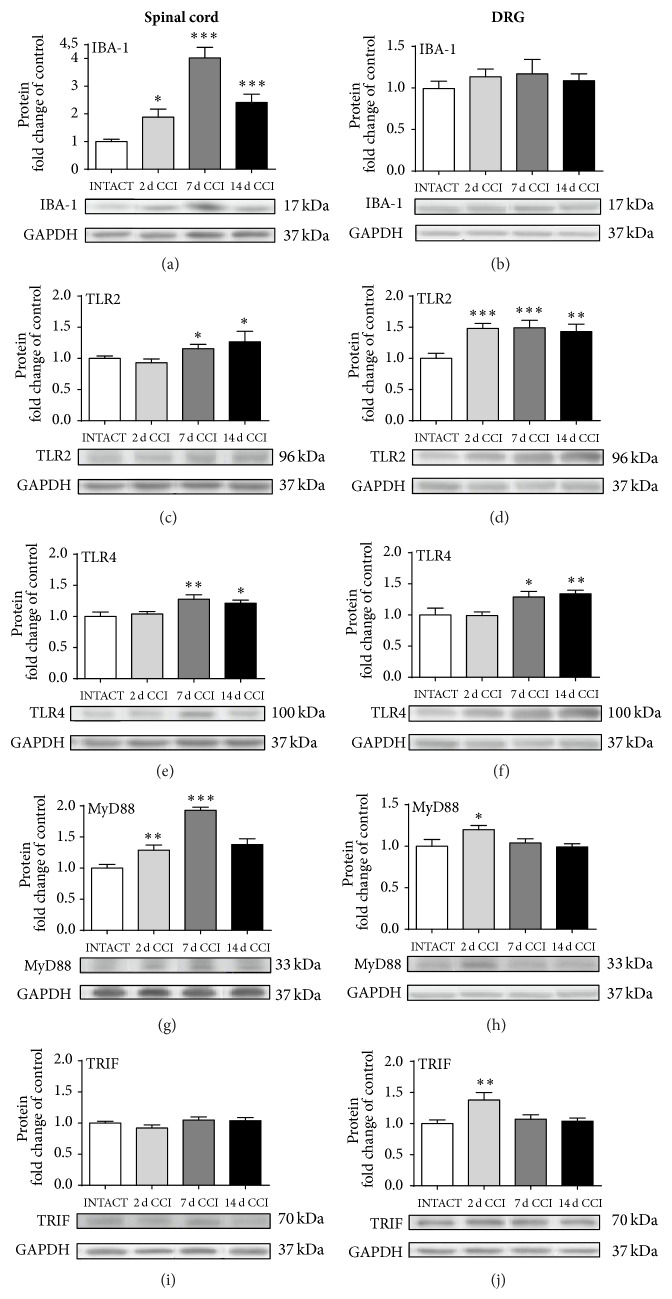
Western blot analysis of IBA-1 (a, b), TLR2 (c, d), TLR4 (e, f), MyD88 (g, h), and TRIF (i, j) protein levels in the ipsilateral dorsal lumbar spinal cord (a, c, e, g, and i) and DRG (b, d, f, h, and j) tissue on 2nd, 7th, and 14th days after chronic constriction injury (CCI) in rats. The data are presented as the mean ± SEM of 4–7 samples per group. Intergroup differences were analyzed using one-way ANOVA followed by Bonferroni's multiple comparisons test. ^*∗*^ *p* < 0.05, ^*∗∗*^ *p* < 0.01, and ^*∗∗∗*^ *p* < 0.001 indicate significant differences versus the INTACT group.

The 88% increase in IBA-1-positive cells was already observed on day 2 in the spinal cord. Even stronger upregulation of 302% was measured on day 7, which slowly decreased to 141% on day 14 after CCI (Figure 3(a)). In the DRG, we did not observe any significant changes in IBA-1 protein after CCI (Figure 3(b)).

The pattern of TLR2 protein level changes showed an increase of 16% and 27% 7 and 14 days after CCI in the spinal cord (Figure 3(c)); in the DRG, additional (48%) upregulation was already observed on day 2, which lasted at a high level (43%) until the 14th day (Figure 3(d)).

Changes in TLR4 protein levels were not observed on day 2 in either the spinal cord or the DRG. However, 28% upregulation was detected on day 7 in the spinal cord and was constant (21%) until day 14 (Figure 3(e)). Similar regulation was observed in the DRG: rises of 29% and 34% on days 7 and 14 after CCI (Figure 3(f)).

An increase in MyD88 protein was already observed in the spinal cord on day 2, with a peak on day 7 (93%); then expression slowly diminished with time, reaching an increase of 38% (Figure 3(g)). In the DRG, 20% upregulation was observed only on day 2 after CCI (Figure 3(h)). Changes in TRIF protein level were not detected in the spinal cord (Figure 3(i)); however, 38% upregulation was detected on day 2 in the DRG (Figure 3(j)).

**Figure 4 fig4:**
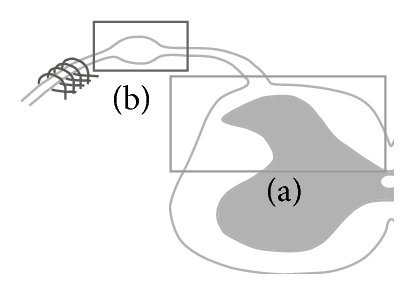
(a) Spinal cord and (b) DRG.

We have not observed significant changes in IBA-1, TLR2, TLR4, MyD88, and TRIF protein levels on the contralateral side of the spinal cord and DRG (Table 1).

### 3.4. *LPS-RS* and* LPS-RS Ultrapure* Administration Attenuated Allodynia and Hyperalgesia, as Measured 2 and 7 Days after CCI

**Figure 5:**
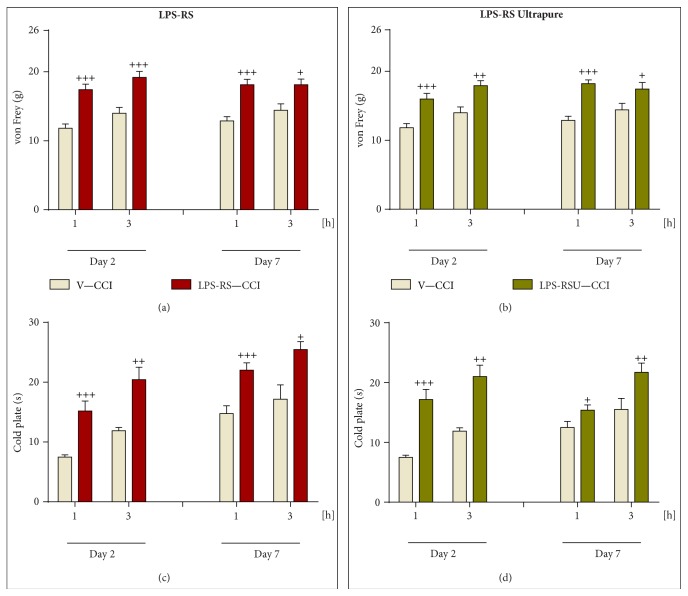
The influence of once daily, repeated intrathecal administration of vehicle (“V”, sterile water),* LPS-RS *[20 *μ*g/5 *μ*L], or* LPS-RS Ultrapure* [“LPS-RSU”, 20 *μ*g/5 *μ*L] on pain behavior, as measured by von Frey's test (mechanical allodynia; (a), (b)) and the cold plate test (thermal hyperalgesia; (c), (d)), 1 and 3 hours after drug administration on 2nd and 7th days after chronic constriction injury (CCI) to the sciatic nerve. The data are presented as the mean ± SEM (10–25 rats per group). Intergroup differences were analyzed using one-way ANOVA followed by Bonferroni's multiple comparisons test. ^+^ *p* < 0.05, ^++^ *p* < 0.01, and ^+++^ *p* < 0.001 indicate significant differences compared with the vehicle-treated, CCI-exposed rat group (V-CCI).

All of the vehicle-treated, CCI-exposed rats revealed neuropathic pain symptoms after surgery. Strong allodynia was measured on days 2 and 7 after injury by von Frey's test (Figures 5(a) and 5(b)), and thermal hyperalgesia was measured by cold plate test (Figures 5(c) and 5(d)). Repeated administration of both drugs:* LPS-RS* and* LPS-RS Ultrapure* [20 *μ*g/5 *μ*L; i.th.], was effective in reducing hypersensitivity, as measured one and three hours after drug administration on days 2 and 7 after injury (Figures 5(a)–5(d)).

### 3.5. Chronic* LPS-RS* or* LPS-RS Ultrapure* Treatment Influences the Analgesic Effects of Single Buprenorphine but Not Morphine Administration, as Measured on Day 7 after CCI

**Figure 6:**
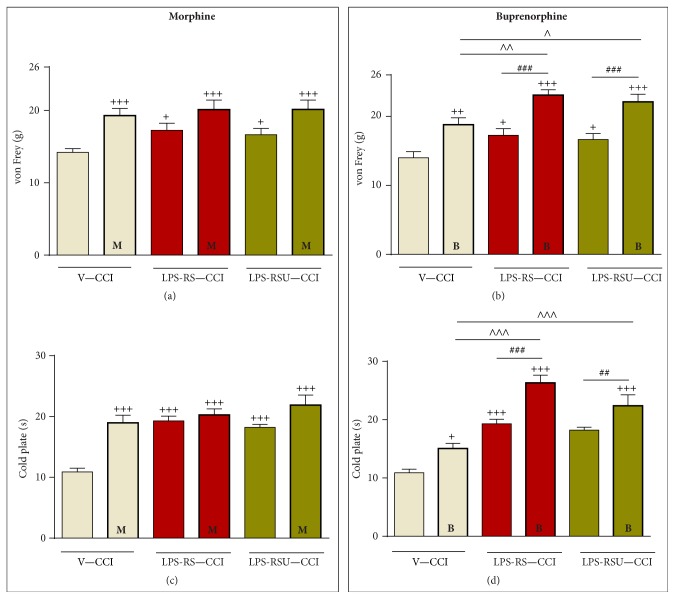
The influence of a single intrathecal administration of vehicle (“V”, sterile water),* LPS-RS *[20 *μ*g/5 *μ*L], or* LPS-RS Ultrapure* [“LPS-RSU” 20 *μ*g/5 *μ*L] on morphine (“M”, 2.5 *μ*g/5 *μ*L; (a), (c)) or buprenorphine (“B”, 2.5 *μ*g/5 *μ*L; (b), (d)) analgesia, as measured by von Frey's test (mechanical allodynia; (a), (b)) and the cold plate test (thermal hyperalgesia; (c), (d)) 7 days after chronic constriction injury (CCI). The data are presented as the mean ± SEM. Intergroup differences were analyzed using one-way ANOVA followed by Bonferroni's multiple comparisons test. ^+^ *p* < 0.05, ^++^ *p* < 0.01, and ^+++^ *p* < 0.001 indicate significant differences compared with the vehicle-treated, CCI-exposed rats (V-CCI); ^#^ *p* < 0.05, ^##^ *p* < 0.01, and ^###^ *p* < 0.001 indicate significant differences between the* LPS-RS* and* LPS-RS Ultrapure *treated CCI-exposed groups after additional vehicle administration comparing to additional morphine or buprenorphine treated CCI-exposed groups; ^∧^ *p* < 0.05, ^∧∧^ *p* < 0.01, and ^∧∧∧^ *p* < 0.001 indicate significant differences between vehicle-treated CCI-exposed groups after morphine or buprenorphine treatment and* LPS-RS/LPS-RSU* opioid treated CCI-exposed groups.

Repeated intrathecal administration of* LPS-RS *and* LPS-RS Ultrapure* [20 *μ*g/5 *μ*L* i.th.;* both] as well as single intrathecal injection of morphine or buprenorphine [2.5 *μ*g/5 *μ*L,* i.th.;* both] on day 7 following CCI attenuated neuropathic pain symptoms in the rats (Figure 6). The analgesic effects of morphine or buprenorphine alone were similar to those of* LPS-RS *and* LPS-RS Ultrapure*, as measured 30 minutes after injection. The morphine/drug combination (2.5 *μ*g of morphine 30 minutes after the administration of* LPS-RS *and* LPS-RS Ultrapure*) did not lead to a more effective analgesic effect in either the von Frey test (Figure 6(a)) or the cold plate test (Figure 6(c)). Interestingly, the buprenorphine/drug combination (2.5 *μ*g of buprenorphine 30 minutes after the administration of* LPS-RS *and* LPS-RS Ultrapure*) led to an enhancement of the effectiveness of the opioid, as measured by von Frey's test (Figure 6(b)) and cold plate test (Figure 6(d)). The effect of buprenorphine was close to cut-off in the injured rats when combined with* LPS-RS* and* LPS-RS Ultrapure*.

## 4. Discussion

Our experiments have completed the data that was already available from different models regarding the contribution of TLR2 and TLR4 in the modulation of neuropathic pain. We show that, under chronic pain conditions, there are time-dependent changes in both the mRNA and protein levels of TLR2 and TLR4 as well as their adaptor molecules (MyD88 and TRIF/TICAM2), which appears parallel to the activation of macrophages/microglial cells. The TLR antagonists* LPS-RS* (TLR2 and TLR4) and* LPS-RS Ultrapure* (TLR4) similarly diminished pain behavior after CCI, suggesting a greater contribution of TLR4 in neuropathy, at least in the rat nerve injury-induced neuropathic pain model. Moreover, this pharmacological interference enhanced buprenorphine's but not morphine's antiallodynic and antihyperalgesic properties under conditions of neuropathic pain.

Existing research therapies against pain seem to have limited effectiveness, partly because they target mainly neurons and do not influence microglial activation. Therefore, in light of our results and available preclinical reports, an exciting alternative of targeting microglial activation is becoming one of the first steps in diminishing the progression of neuropathic pain. Understanding the relationship between microglia and TLRs may help in developing new targets for drugs. Solid evidence indicates the critical involvement of microglia in neuropathy, reinforcing the idea that these cells not only are a structural support for neurons but also contribute significantly to their function [16, 26, 29, 30]. The signals that induce microglial activation in response to nerve injury remain incompletely clarified. Among the various receptors expressed on microglia, the Toll-like family, especially subtypes 2 and 4, are a possible answer to that problem because they act as a link between microglial activation and nerve injury and play a crucial role in the development of neuropathic pain symptoms [31, 32].

Our studies were performed using the neuropathic pain model developed by Bennett and Xie [23]. We observed that mechanical allodynia and thermal hyperalgesia develop as soon as 2 days after sciatic nerve injury. Our qRT-PCR analysis revealed simultaneous upregulation in TLR4 expression in the lumbar (L4–L6) dorsal spinal cord and DRG, which was significant until day 14 after CCI in the Wistar rats. Similar results were observed by Wu et al. [33] in Sprague-Dawley rats at the spinal level on day 7 and by other laboratories in diabetic mouse models [34, 35]. Our results showed the enhanced expression of TLR2 in the spinal cord and DRG on days 7 and 14 after CCI. Similar results were obtained by others using a mouse CCI model [36]. MyD88 mRNA levels remained elevated throughout the whole time course in the spinal cord and DRG. There are no corresponding data for neuropathic pain; however, similar results were obtained in the Sprague-Dawley rat model of irritable bowel syndrome (IBS) hypersensitivity [37]. TICAM2 expression was significantly enhanced in the spinal cord, with a peak on day 7 after surgery; however, in contrast to the protein results, there was no difference from baseline detected in the DRG. There are no data available regarding TICAM2 or TRIF regulation in chronic pain states.

As we have shown, the qRT-PCR analysis of TLR2, TLR4, and MyD88 [34, 35, 37] expression was carried out by several laboratories, although changes in their protein levels are poorly examined under neuropathic pain conditions. To date, the only published data available regarding protein levels of TLR4 and its signaling molecules in neuropathy were provided by Li et al. [12] in a paclitaxel-related chemotherapy-induced peripheral neuropathy model. We have shown using Western blot analysis in the CCI model that TLR4 protein levels are upregulated in the spinal cord on days 7 and 14 after CCI. Similar spinal regulation was published recently in the CIPN model by Li's group, who reported significant upregulation of TLR4 protein levels in parallel to pain development, as measured in the CIPN Sprague-Dawley rat model [11]. In the DRG, however, the changes are quite opposite because elevation is observed during the early stages of neuropathy development and in our model, from day 7, when neuropathic pain has already developed. We are the first to report that TLR2 protein levels, similar to TLR4 protein levels, are also upregulated in the lumbar spinal cord during the second week of pain development. The importance of TLR2 was already suggested by Shi et al. [36], who reported that, in TLR2 KO mice, nerve injury-induced thermal hyperalgesia was completely abolished. This finding is contrary to that observed in wild-type mice, in which mechanical allodynia was partially reduced. Shi et al. suggested that TLR2 is necessary for the development of neuropathic pain and that its contribution is more important in thermal hypersensitivity than in mechanical allodynia. In our experiment, TLR2 and TLR4 were also upregulated in DRG tissue at all of the time points measured after CCI-induced neuropathy. This implies that the only change that can be observed in the DRG occurs at the beginning of neuropathy progression, so it can be assumed that the response of TLR4 to the injury state in the DRG is faster than that of TLR2. However, a distinction between the possible contributions of these two receptors to neuropathic pain cannot be ascertained based on the depicted results, and this issue needs future study. We decided to investigate the changes in the levels of the MyD88 and TRIF adaptor molecules to verify if there is difference between TLR2 (connected to MyD88) and TLR4 (connected to both MyD88 and TRIF) activation. Our data suggest that CCI induced a strong and gradual increase in MyD88 protein until day 7, with a slight, though not significant, reduction on the 14th day. Other results reported in the CIPN model showed no significant changes in spinal MyD88 protein [12]. In the DRG, as we believe, both our results and the results of Li et al. [12] are consistent and show an increase in MyD88 in the early stage (on the 2nd day) of neuropathy development. The results obtained in our experiments indicate no spinal changes in TRIF protein over the whole time course and a short-term rise in the DRG in the early stage of neuropathy development (on the 2nd day), which is in agreement with the results obtained by Li et al. [12]. Our results suggest that, in the spinal cord, MyD88 (TLR2 and TLR4) is activated but TRIF (TLR4) is not. Because the biochemical studies did not allow us to differentiate the role of these two receptors in neuropathic pain, we employed pharmacological studies using a TLR4-specific antagonist (*LPS-RS Ultrapure*) and for comparison a TLR4- and TRL2-specific antagonist (*LPS-RS*). By our experiments, we have shown that, surprisingly, both substances similarly diminished the thermal hyperalgesia and tactile allodynia in our model of neuropathic pain.

Our results obtained after* LPS-RS *administration are in agreement with those reported using different animal models, showing its beneficial effects in other models, for example, the Sprague-Dawley rat paclitaxel-induced neuropathy model [12], the Wistar rat cancer-induced bone pain model [11], the* C57Bl/6* mouse inflammatory arthritis pain model [10], and the Sprague-Dawley rat nerve injury-induced model [13]. Our results also show that chronic intrathecal administration of* LPS-RS* diminished neuropathic pain induced by mechanical nerve injury. To date, there have not been any reports that* LPS-RS Ultrapure* has any influence on the maintenance of neuropathic pain in any model; thus, we are the first to report that specific antagonism of TLR4 is enough to produce an analgesic effect. Taking this into consideration, we assumed that antagonism of both TLR2 and TLR4 by* LPS-RS* would have diminished more of the neuropathic pain symptoms than antagonism of only TLR4 by the specific antagonist* LPS-RS Ultrapure*. However, our data suggest that TLR4 makes a greater contribution to neuropathy development and maintenance, at least in the rat nerve injury-induced neuropathic pain model.

Because it has been already published that antagonism of TLR4 enhances morphine analgesia in various contexts, our results seem to shed light on the extremely important passage through this theory. Namely, our data indicate that both* LPS-RS* and* LPS-RS Ultrapure* enhance the effectiveness of buprenorphine but not morphine. Most of the studies in INTACT animals reported to date refer to the attenuation of morphine tolerance by TLR4 antagonism in both rat and mouse models [18–20]. In 2013, Eidson and Murphy [17] reported a complex study using the CFA model in male Sprague-Dawley rats, which showed that cumulative doses of morphine along with a single systemic (*s.c.*) injection of (+)-naloxone resulted in the enhancement of opioid analgesia. Moreover, TLR4 antagonism directly in the PAG showed a similar effect, but, without morphine, there was no analgesia reported [17]. Although many hypotheses have been proposed to date [38], no strict evidence has been published demonstrating that TLR4 antagonism* in vivo* could actually be effective alone and moreover enhance opioid effectiveness under neuropathic pain conditions. Although there are works suggesting that TLR4 antagonism potentiates the analgesic efficacy of morphine, the studies were performed in INTACT animals, not in a neuropathic pain model (so in the absence of pain) [39, 40]. Knowing that a single, acute injection of an effective dose [2.5 *μ*g;* i.th.*] of morphine or buprenorphine attenuates hypersensitivity in neuropathic rats, here, we report that although the TLR4 antagonists actually enhanced buprenorphine's analgesic effect, in contrast, the effect of morphine was not enhanced. Morphine suppresses neuropathic pain via opioid receptors, while buprenorphine also activates nociceptin/orphanin FQ peptide (NOP) receptors [41]. Preliminary data published to date suggests there is a link between TLR4 activation by an exogenous ligand (LPS) and NOP upregulation because they indicate that antagonism of TLR4 also attenuates the enhancement of NOP levels [42]. To test this hypothesis, we conducted a biochemical experiment in which we compared levels of NOP protein in groups treated with LPS-RS/LPS-RS Ultrapure/vehicle to levels in INTACT animals. We observed significant elevation of nociceptin receptor expression after injury (vehicle-treated) and attenuation of this effect after drug treatment (*data not shown*). Our observation that the effect of buprenorphine can be enhanced (in contrast to morphine) by antagonism of TLR2 or TLR4 needs further evaluation because it could explain the possibility of buprenorphine opioid rotation after the development of morphine tolerance.

## 5. Conclusion

Under conditions of neuropathic pain, we have measured upregulation of CD40, TLR2, TLR4, MyD88, and TICAM2 mRNA in the spinal cord and/or DRG using qRT-PCR method. The Western blot technique revealed upregulation of IBA-1, TLR2, TLR4, MyD88, and TRIF protein levels in the spinal cord and/or DRG. Our data suggest that both TLR2 and TLR4 may play a significant role in neuropathic pain, in light of their upregulation over the course of chronic pain, which could be linked to the activation of microglia and other IBA-1/CD40-positive cells that was also observed. Blockade of TLR2 and TLR4 produced analgesia and moreover enhanced the effectiveness of buprenorphine. The graphical abstract of our main results is available in the Supporting Information. Understanding the link between microglia and TLRs may help in developing new targets for pharmacotherapy. Depicted results may have great importance and possible clinical implications in neuropathy therapies in human due to their high conservatism of TLRs between species.

## Supplementary Material

Protein and mRNA levels of TLR2, TLR4 and their adaptor molecules: MyD88 and TRIF are upregulated in spinal cord and/or DRG as measured in three time points during 14-day course of neuropathy development. Pharmacological blockade of Toll-like receptors (TLR2, TLR4) with endogenous antagonists - LPS-RS and LPS-RS Ultrapure, has evoked analgesia in rat CCI neuropathic pain model and potentiated buprenorphine antinociceptive effect.

## Figures and Tables

**Table 1:** Western blot analysis of IBA-1, TLR2, TLR4, MyD88, and TRIF protein levels in the contralateral dorsal lumbar spinal cord and DRG tissue on 7th day after chronic constriction injury (CCI) in rats. The results are not statistically significant and are presented as the mean ± SEM of 4–7 samples per group (see also Figure 4).

Protein level	7th day after CCI			
	Dorsal lumbar section, contralateral side			
	INTACT		Vehicle-CCI	
Spinal cord	IBA-1	1.00 ± 0.07	IBA-1	1.12 ± 0.19
	TLR2	1.00 ± 0.13	TLR2	1.17 ± 0.08
	TLR4	1.00 ± 0.06	TLR4	1.11 ± 0.13
	MyD88	1.00 ± 0.05	MyD88	1.09 ± 0.06
	TRIF	1.00 ± 0.04	TRIF	0.99 ± 0.06

DRG	IBA-1	1.00 ± 0.10	IBA-1	1.02 ± 0.08
	TLR2	1.00 ± 0.08	TLR2	0.81 ± 0.11
	TLR4	1.00 ± 0.16	TLR4	0.98 ± 0.07
	MyD88	1.00 ± 0.11	MyD88	0.98 ± 0.05
	TRIF	1.00 ± 0.03	TRIF	1.00 ± 0.08
