# Supplementary material for: Blockade of Toll-Like Receptors (TLR2, TLR4) Attenuates Pain and Potentiates Buprenorphine Analgesia in a Rat Neuropathic Pain Model
Source: Neural Plast. 2015 Dec 29;2016:5238730. doi: 10.1155/2016/5238730 (PMC4709736; doi:10.1155/2016/5238730)

BLOCKADE OF TOLL-LIKE RECEPTORS (TLR2, TLR4) ATTENUATES PAIN AND POTENTIATES BUPRENORPHINE ANALGESIA IN A RAT NEUROPATHIC PAIN MODEL

UNDER NEUROPATHIC PAIN WE HAVE OBSERVED:

- **Enhancement in TLR2 & TLR4, MyD88 and TRIF expression in the spinal cord and/or DRG**
- **Upregulation of TLR2 & TLR4, MyD88 and TRIF protein levels in the spinal cord and/or DRG**
- **Analgesic effects of TLR antagonists: *LPS-RS* (TLR2 & TLR4) or *LPS-RS Ultrapure* (TLR4)**
- **Improvement of buprenorphine analgesia by LPS-RS or LPS-RS Ultrapure**

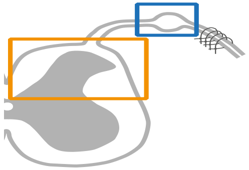

	mRNA			PROTEIN		
	DAY 2	DAY 7	DAY 14	DAY 2	DAY 7	DAY 14
**TLR2**	— / —	↑↑ / ↑↑↑	↑↑↑ / ↑	— / ↑↑↑	↑ / ↑↑↑	↑ / ↑↑
**TLR4**	↑↑↑ / ↑↑	↑↑↑ / ↑	↑↑↑ / ↑	— / —	↑↑ / ↑	↑ / ↑↑
**MyD88**	↑↑↑ / ↑↑↑	↑↑↑ / ↑↑↑	↑↑↑ / ↑↑↑	— / ↑	↑↑↑ / —	— / —
**TRIF / Ticam2**	↑↑ / —	↑↑↑ / —	↑↑↑ / —	↑↑ / ↑↑	— / —	— / —

→ SPINAL CORD → DRG

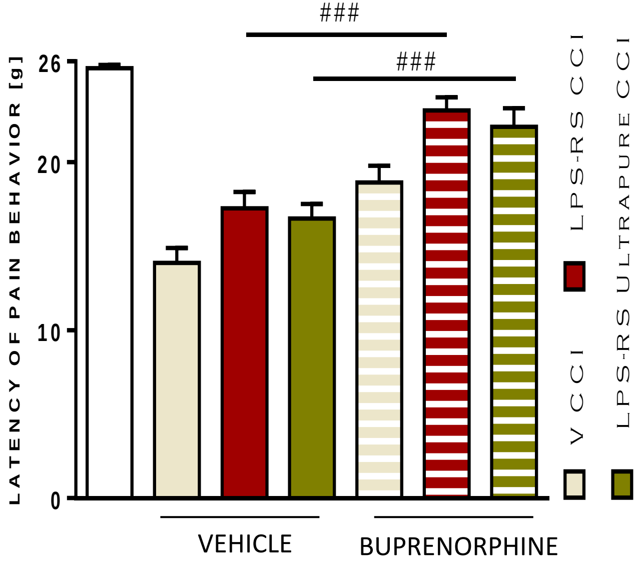

Supplement: Supplementary file 1 — Protein and mRNA levels of TLR2, TLR4 and their adaptor molecules: MyD88 and TRIF are upregulated in spinal cord and/or DRG as measured in three time points during 14-day course of neuropathy development. Pharmacological blockade of Toll-like receptors (TLR2, TLR4) with endogenous antagonists - LPS-RS and LPS-RS Ultrapure, has evoked analgesia in rat CCI neuropathic pain model and potentiated buprenorphine antinociceptive effect. [file 5238730.f1.pdf]
